# Comparison of a Full Food-Frequency Questionnaire with the Three-Day Unweighted Food Records in Young Polish Adult Women: Implications for Dietary Assessment

**DOI:** 10.3390/nu5072747

**Published:** 2013-07-19

**Authors:** Joanna Kowalkowska, Malgorzata A. Slowinska, Dariusz Slowinski, Anna Dlugosz, Ewa Niedzwiedzka, Lidia Wadolowska

**Affiliations:** 1 Department of Human Nutrition, University of Warmia and Mazury, Słoneczna 44a, Olsztyn 10-718, Poland; E-Mails: malgorzata.slowinska@uwm.edu.pl (M.A.S.); ewa.niedzwiedzka@uwm.edu.pl (E.N.); lidia.wadolowska@uwm.edu.pl (L.W.); 2 Chair of Geotechnics and Road Engineering, University of Warmia and Mazury, Prawocheńskiego 19, Olsztyn 10-720, Poland; E-Mail: dariusz.slowinski@uwm.edu.pl; 3 Chair and Department of Nutrition and Dietetics, Nicolaus Copernicus University, Collegium Medicum, Dębowa 3, Bydgoszcz 85-626, Poland; E-Mail: anna.walus@wp.pl

**Keywords:** Polish FFQ, 3-day food records, energy intake, nutritional value, diet, women

## Abstract

The food frequency questionnaire (FFQ) and the food record (FR) are among the most common methods used in dietary research. It is important to know that is it possible to use both methods simultaneously in dietary assessment and prepare a single, comprehensive interpretation. The aim of this study was to compare the energy and nutritional value of diets, determined by the FFQ and by the three-day food records of young women. The study involved 84 female students aged 21–26 years (mean of 22.2 ± 0.8 years). Completing the FFQ was preceded by obtaining unweighted food records covering three consecutive days. Energy and nutritional value of diets was assessed for both methods (FFQ-crude, FR-crude). Data obtained for FFQ-crude were adjusted with beta-coefficient equaling 0.5915 (FFQ-adjusted) and regression analysis (FFQ-regressive). The FFQ-adjusted was calculated as FR-crude/FFQ-crude ratio of mean daily energy intake. FFQ-regressive was calculated for energy and each nutrient separately using regression equation, including FFQ-crude and FR-crude as covariates. For FR-crude and FFQ-crude the energy value of diets was standardized to 2000 kcal (FR-standardized, FFQ-standardized). Methods of statistical comparison included a dependent samples *t*-test, a chi-square test, and the Bland-Altman method. The mean energy intake in FFQ-crude was significantly higher than FR-crude (2740.5 kcal *vs.* 1621.0 kcal, respectively). For FR-standardized and FFQ-standardized, significance differences were found in the mean intake of 18 out of 31 nutrients, for FR-crude and FFQ-adjusted in 13 out of 31 nutrients and FR-crude and FFQ-regressive in 11 out of 31 nutrients. The Bland-Altman method showed an overestimation of energy and nutrient intake by FFQ-crude in comparison to FR-crude, e.g., total protein was overestimated by 34.7 g/day (95% Confidence Interval, CI: −29.6, 99.0 g/day) and fat by 48.6 g/day (95% CI: −36.4, 133.6 g/day). After regressive transformation of FFQ, the absolute difference between FFQ-regressive and FR-crude equaled 0.0 g/day and 95% CI were much better (e.g., for total protein 95% CI: −32.7, 32.7 g/day, for fat 95% CI: −49.6, 49.6 g/day). In conclusion, differences in nutritional value of diets resulted from overestimating energy intake by the FFQ in comparison to the three-day unweighted food records. Adjustment of energy and nutrient intake applied for the FFQ using various methods, particularly regression equations, significantly improved the agreement between results obtained by both methods and dietary assessment. To obtain the most accurate results in future studies using this FFQ, energy and nutrient intake should be adjusted by the regression equations presented in this paper.

## 1. Introduction

Adequate assessment of food intake and changes in dietary habits plays a highly significant role in research on health and nutrition [1]. All food assessment methods are limited by specific errors [1,2,3]. Therefore, the results obtained from the same respondents, but with other methods, can differ and make their interpretation difficult.

The food frequency questionnaire and the food record are some the most often used methods in dietary research [1,4]. The food record is a prospective method, independent of the respondent’s memory and usually covers several consecutive days [1,2]. The limitations of this method include, among others, not taking into consideration the long-term variety of consumption, possible changes in dietary habits, and simplification of menus resulting from a significant burden on the respondents [1,2,4,5,6,7,8]. The size of error depends on the time of examination, and on the characteristics of the respondent (e.g., underestimation of intake is more common in women or persons after a slimming diet) [1,4,5,8]. It is not recommended to extend the period of examination for more than seven consecutive days, as this method excessively involves the respondent [4]. Generally, the food record method provides relatively accurate data concerning intake of food and nutrients. Therefore, other nutrition assessment methods are often compared to it.

The food frequency questionnaire is often used to assess the relationship between the diet and the disease [1,2,4]. It is a retrospective method, based on the memory of the respondent, and most often concerns a longer period of time, e.g., one month or year, and therefore represents a habitual diet [1,2]. It requires a larger sample size, but usually involves the respondent less than most dietary assessment methods. This is a method of a lower accuracy, which is rather suitable to establishing a personal ranking according to food or nutrient intake than to assess the precise level of intake [1,2,3,4,5,9]. Possible errors include omission or addition of food, or inadequate assessment of the frequency and amount of consumed products [1,2]. Food frequency questionnaires require validation, constant updating of food list (new products, technologies), and adjustment to the purpose of research, region, or age of respondents [1,10]. Consequently, the quality of the data largely depends on the quality of the food frequency questionnaire.

Both dietary assessment methods are characterized by difficulties in accurate estimation of portion sizes, which may lead to overestimation or underestimation of food intake depending on the specificity of the method [4,5,10]. Food frequency questionnaires usually overestimate food intake as compared to other nutritional assessment methods [4], which leads to overestimating energy and nutritional value of diets. Numerous studies demonstrated a higher energy and nutritional value of diets if a food frequency questionnaire was used in comparison to food records or 24 h interviews [6,11,12,13,14,15,16]. Single studies demonstrated a lack of significant difference in energy intake between food frequency questionnaire and food records [7], or an underestimation of energy and nutrient intake by the food frequency questionnaire [17].

In order to reduce the effect of overestimating or underestimating food intake on the nutritional value assessment of diets, energy adjustment can be used [4,6,8,18,19,20]. Energy adjustment allows analyzing the composition of the diet regardless of errors, e.g., inadequate estimation of the portion size. Energy adjustment can also distort the true value of the diet, resulting from the intake of some nutrients, e.g., fat [4]. The literature describes several methods of adjustment of energy and nutrient intake [4,8,20]. The choice of an adequate approach depends on the aim of research.

In Poland, little research has been carried out involving the comparison of results obtained from the same respondents by the food frequency questionnaire or other methods of dietary assessment [3,21,22]. The wide application of the food record and food frequency questionnaire in research concerning nutrition and health justifies interest in their methodological aspects. Literature covering this field is very extensive, although it concerns food frequency questionnaires developed in other countries [6,7,9,11,13,15,16,23]. Taking into account the national specificity, it is not known to what extent the results of the research in which various methods were used can be jointly used to interpret the effects of nutrition on health. The aim of this study was to compare the energy and nutritional value of diets determined by the full food frequency questionnaire [21] and obtained by using three-day food records of young women.

## 2. Experimental Section

The first study was approved by the Bioethics Committee of the Regional Medical Chamber in Olsztyn in 2001, and the second study was approved by the Bioethics Committee of the Faculty of Medical Sciences, University of Warmia and Mazury in Olsztyn in 2010.

### 2.1. Participants

The research was carried out in 2010 among female students of the Faculty of Food Science of the Warmia and Mazury University in Olsztyn (*n* *=* 23) and in 2003–2004 among female students of the Faculty of Food Science of the Warmia and Mazury University in Olsztyn and female students of Collegium Medicum of the Nicolaus Copernicus University in Bydgoszcz (*n =* 61). After the findings accumulated in the years 2003–2004 were analyzed, it was decided to increase the size of the sample. Another analysis confirmed the earlier conclusions and did not reveal any differences between the 2003–2004 findings and those of 2010. This shows that the combined analysis of the data is justified. In total, the research involved 84 women aged 21 to 26 years (mean of 22.2 ± 0.8 years, Table 1).

**Table 1:** Sample characteristics (*n* 84).

Category	Sample size (*n*)	Percentage of women (%)
Age	–	22.2 ± 0.8 # (21–26)
Place of residence		
Village	23	27
town (<50,000 inhabitants)	22	26
small city (50,000–100,000 inhabitants)	21	25
large city (>100,000 inhabitants)	18	21
Self-assessment of economic situation		
below average	1	1
Average	79	94
above average	4	5
Self-assessment of lifestyle		
active	0	0
quite active	10	12
little active	30	36
a sedentary lifestyle	44	52
Self-assessment of health status		
very good	11	13
Good	66	79
quite good	6	7
Poor	1	1
Suffering from chronic diseases	5	6
Time spent in front of TV		
≥1 h/day	8	10
2–3 h/day	34	40
≤0 h/day	42	50
Self-declared to be following a diet		
no diet	58	69
yes, taking care of a slim figure (not overeating)	19	23
yes, low-energy diet	2	2
yes, low-fat diet	1	1
yes, other diet	4	5
Self-declared change in dietary habits during a year (yes)	72	86
Number of meals eaten per day		
2	2	2
3	33	39
4	36	43
5	13	15

# mean and standard deviation; () in brackets given minimum-maximum range of age.

### 2.2. Dietary Assessment Methods

The content of energy and following nutrients in the diet of respondents was determined using three-day unweighted food records (FR) and the food frequency questionnaire (FFQ): total protein, animal protein, vegetable protein, fat, saturated fatty acids (SFA), monounsaturated fatty acids (MUFA), polyunsaturated fatty acids (PUFA), cholesterol, carbohydrates, fiber, water, sodium, potassium, calcium, phosphorus, magnesium, iron, zinc, copper, Vitamin A, retinol, sterol, car Vitamin D, Vitamin E, Vitamin B_1_, Vitamin B_2_, niacin, Vitamin B_6_, folic acid, Vitamin B_12_, and Vitamin C.

A fully self-administered FFQ was used, for which the internal compatibility was examined [21]. A high reproducibility of results obtained by the FFQ regarding the frequency and amount of usually consumed food was found. The questionnaire was considered an accurate measurement tool. The FFQ was developed to study the eating habits of young women in relation to diet-related diseases.

The FFQ contained a list of 165 products and dishes, which represented all food groups. The questionnaire covered the entire Polish diet and contained questions about many of the most popular Polish dishes, consumed anywhere (at home or restaurants). The FFQ described food consumption during the last year. For each product/dish, the respondents freely determined their usual portion size using an “Album of photographs of food products and dishes” [24], and usual frequency of intake during: a day, a week, a month, or a year. Additionally, they could choose the answers: *never*, *I don’t know how often*, *I don't know if I had*. The questionnaire took into account the seasonal character of consumption concerning some vegetables (e.g., cucumbers, tomatoes), fruits (e.g., plums, strawberries), and ice-cream. An algorithm was developed using typical food recipes, which was used to convert dishes to the intake of single products.

Descriptive characteristics in the FFQ included some questions regarding to socioeconomic situation, health, and lifestyle (shown in Table 1):

– place of residence: (i) village; (ii) town (<50,000 inhabitants); (iii) small city (50,000–100,000 inhabitants); (iv) large city (>100,000 inhabitants);
– self-assessment of the economic situation: (i) below average; (ii) average; (iii) above average;
– self-assessment of the lifestyle: (i) active (intensive physical activity every day); (ii) quite active (intensive physical activity 2–3 times per week); (iii) little active (intensive physical activity once a week); (iv) a sedentary lifestyle (no intensive physical activity);
– self-assessment of the health status: (i) very good; (ii) good; (iii) quite good; (iv) poor;
– suffering from chronic diseases: yes, no;
– time spent in front of TV: (i) ≥4 h/day; (ii) 2–3 h/day; (iii) ≤1 h/day;
– self-declared to be following a diet: (i) no diet; (ii) yes; taking care of a slim figure (not overeating); (iii) low-energy diet; (iv) low-fat diet; (v) other diet (e.g., diabetes, easily digestible, vegetarian);
– self-declared change in dietary habits during a last year: yes, no;
– number of meals eaten per day–the respondents could freely indicate the number of meals usually eaten per day.

Filling-in the FFQ was preceded by performance of the food records. Respondents were precisely instructed and presented with examples on how to complete the questionnaire and perform unweighted food records.

Food records covered three consecutive days, two weekdays and one day of the weekend. Respondents recorded the type and amount (in household measures) of consumed products, dishes and beverages. The amount of food consumed was determined with the use of an “Album of photographs of food products and dishes” [24], and expressed in grams.

All food records and completed questionnaires were checked in terms of their careful performance and doubts were explained in a direct verifying interview. Interviews of three persons were rejected due to errors, e.g., incomplete or incorrectly completed questionnaires (3.4% of the initial sample). Afterwards, using Polish “Food composition tables” [25], the mean daily energy and selected nutrient content in a diet was calculated for each respondent for both methods separately. Use of nutrient supplementation was not taken into consideration.

### 2.3. Adjustment of the Intake of Energy and Nutrients Obtained from FFQ

Differences were found in the average energy and nutritional value of diets determined with both methods. Therefore, the mean daily intake of energy and nutrients obtained by means of FFQ was adjusted, aiming at obtaining values as close as possible to those obtained by a three-day unweighted food record (reference method). Such a procedure was adopted on the basis of literature data [4,12,13,14,15,20] and own experience, which prove that the food frequency questionnaires tend to overestimate the intake. It was recognized that the food record can provide more precise estimates, despite a shorter time of examination (three days) [4,10,15]. Unadjusted energy and nutrient intake obtained by the food frequency questionnaire was marked as FFQ-crude, and obtained by the food record was marked as FR-crude. As a result of adjustment of energy and nutrient intake determined by the FFQ-crude, the following data were obtained:

– FFQ-adjusted—mean daily intake of energy and nutrients obtained after modification of FFQ-crude using a beta-coefficient equalling 0.5915 (authors’ suggestion), which was calculated as the FR-crude/FFQ-crude ratio of mean daily energy intake. This is a simple way of adjusting the energy and nutrient intake at the group level as proposed by the authors for a quick and effective approximation of the results obtained from the FFQ-crude to the results from the unweighted food record (FR-crude).
– FFQ-regressive—mean daily intake of energy and nutrients obtained after modification of FFQ-crude using regression analysis [26]; separate regression equations were determined for energy and each nutrient.

FFQ-standardized and FR-standardized were determined by converting, for each respondent, the mean daily nutrient intake to 2000 kcal of the energy value of the diet.

### 2.4. Nutritional Assessment According to Dietary Recommendation

The mean daily intake of energy and nutrients according to FR-crude, FFQ-crude, FFQ-adjusted, and FFQ-regressive was compared to Polish nutrition standards [27]. *Z*-values of individual intake of energy and nutrients were calculated and compared to Polish dietary recommendations–estimated average requirement (EAR) or adequate intake (AI) [27,28]. Fiber and cholesterol intake was compared to Polish and WHO recommendations [27,29]. The cut-off value for fiber was 25 g/day and 300 mg/day for cholesterol. On the basis of the characteristics of the sample (Table 1), low physical activity of respondents (PAL = 1.6) was assumed for the calculations. While calculating *z*-values of individual intake for each method, an appropriate number of days were taken into account, *i.e.*, 365 days for FFQ and three days for FR.

The percentage of women who met Polish recommendations for energy and nutrient intake was calculated using the probability method [27]. The established cut-off points (*i.e.*, *z*-values of individual energy and nutrient intake <−1 SD or >1 SD) produced conclusions with a probability of 0.85 [27,28]. Respondents with *z*-values of individual energy and nutrient intake below −1 SD were defined as “women who failed to meet dietary recommendations”, while those above 1 SD were defined as “women who met dietary recommendations”. Between <−1 SD and >1 SD is an energy and nutrient intake level interpreted as “neither met nor does not meet energy and nutrient intake recommendations” [27].

### 2.5. Statistical Analysis

For energy and nutrient intake obtained from food records and FFQ, both before and after adjustment for energy and nutrient intake, the normality of distribution was checked by a Shapiro-Wilk test [26]. Since the distribution of many variables was not compatible with normal distribution, a logarithmic transformation of the data was conducted in order to obtain the normal distribution of variables. The mean values of energy and nutrient intake determined by the FR and the FFQ were compared by a dependent-samples *t*-test.

For each respondent, the FFQ/FR ratio was calculated, *i.e.*, the quotient of mean daily energy (or nutrient) intake determined by FFQ and FR, and expressed as a percentage value. Values of the ratio above 100% were interpreted as overestimation of the intake of a nutrient by FFQ in comparison to FR, and values below 100% were interpreted as underestimation.

The percentages of women in three intake categories (based on *z*-values of energy and nutrient intake) were compared according to: (i) FR-crude and FFQ-crude; (ii) FR-crude; and FFQ-adjusted; (iii) FR-crude and FFQ-regressive by chi-square test.

An evaluation was conducted of the compatibility of respondents’ classification into: (i) the same intake category in FR and FFQ (*i.e.*, FR-crude and FFQ-crude_,_ FR-crude and FFQ-adjusted, FR-crude and FFQ-regressive); (ii) a lower intake category in FFQ than in FR; (iii) a higher intake category in FFQ than in FR.

The Bland-Altman method was used to assess the agreement between the results obtained with both methods [30]. On the basis of energy and nutrient intake obtained by the FFQ and the food record, the following values were calculated: mean absolute difference in nutrient intake in both methods (AD_mean_, formula 1), mean intake of the nutrient for both methods (x_both methods_, formula 2), limits of agreement (LOA, formula 3), and variation coefficient (VC, formula 4).

AD = *x*_FFQ_ − *x*_FR_ (1)

*x*_both methods_ = (*x*_FFQ_ + *x*_FR_)/2 (2)

LOA = AD_mean_ ± 1.96 × SD_difference_ (3)

VC (%) = (SD_difference_/*x*_both methods_) × 100% (4)

where: AD—absolute difference in nutrient intake in both methods (calculated for each respondent), *x*_FFQ_—mean daily energy (or nutrient) intake in FFQ, x_FR_—mean daily energy (or nutrient) intake in the food record, SD_difference_—standard deviation of the absolute difference in nutrient intake in both methods.

The Bland-Altman index (%) was calculated as a percentage of persons beyond the limits of agreement (LOA). Good reproducibility of the measurement is proved by a minimum of 95% differences within the ± 2SD limits, which corresponds to the Bland-Altman index amounting to no more than 5% [30,31].

While interpreting the results, particular importance was given to the Bland-Altman method, which is recommended for comparison of methods and recognized as the “gold standard” [10,23].

Statistical analysis was performed using Statistica PL version 10.0 by StatSoft.

## 3. Results

### 3.1. Food Records (FR-Crude) *vs.* Food Frequency Questionnaire (FFQ-Crude)

The mean intake of energy and nutrients determined by FFQ-crude was significantly higher than that determined by FR-crude, except for cholesterol (Table 2). The mean energy value of the diet in FFQ-crude was 2740.5 kcal (95% CI 2501.9, 2979.1 kcal) and in FR-crude 1621.0 kcal (95% CI 1488.9, 1753.0 kcal). The FFQ-crude/FR-crude ratio for energy was 179% on average, while for the nutrients it ranged, on average, from 140% for sodium to 4449% for Vitamin B_12_ (Table 3).

**Figure 1:**
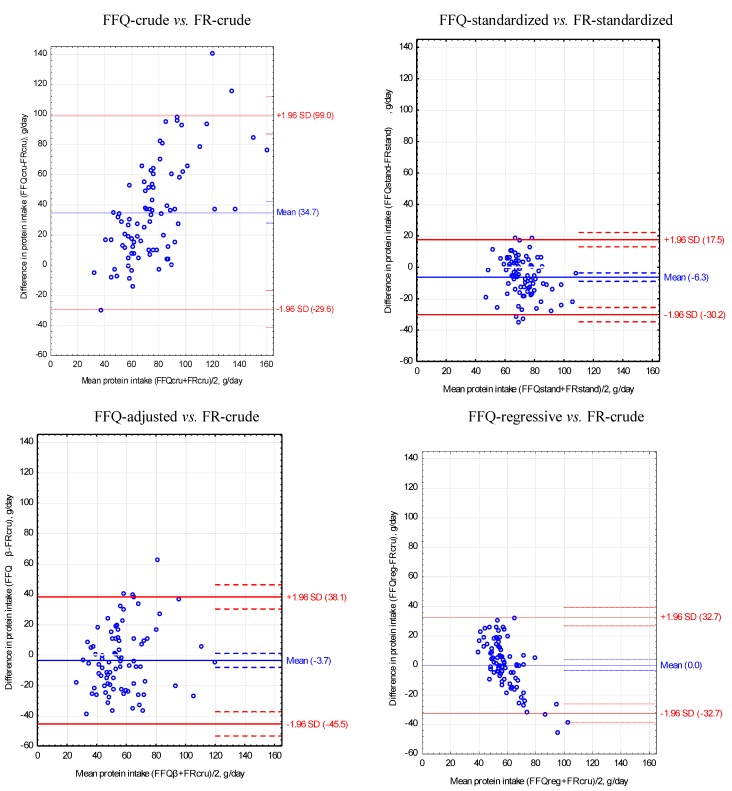
Bland-Altman plots to assess agreement between the food frequency questionnaire (FFQ) and three-day unweighted food record (FR) for protein intake in 84 women.

The Bland-Altman index was not higher than 5% for sixteen nutrients: fiber, potassium, calcium, phosphorus, zinc, copper, Vitamin B_1_, total protein, SFA, Vitamin A, retinol, Vitamin D, Vitamin B_2_, Vitamin B_6_, niacin, and folic acid (Table 3, Figure 1). The lowest values of Bland-Altman index were found for niacin and folic acid (2% each). A variation coefficient (VC) below 50% was obtained for energy and fifteen nutrients. The lowest values of VC were obtained for energy (42%), total protein and carbohydrates (43% each), and the highest for retinol (170%), Vitamin D (124%), and Vitamin B_12_ (105%).

The distributions of the percentage of women in intake categories according to FR-crude and FFQ-crude were significantly different for energy and for four nutrients (PUFA, fiber, potassium, and calcium) (Table 4). A higher number of women who met dietary recommendations were found in FFQ-crude than in FR-crude (55% *vs.* 2%, respectively).

Most women were classified to a higher intake category by FFQ-crude than by FR-crude for energy and 20 out of 22 nutrients (Appendix B). For example, for energy, 26% women were classified to the same intake category by FFQ-crude and FR-crude, 18% women were classified to a lower intake category and 56% women were classified to a higher intake category by FFQ-crude than FR-crude. More women were classified to a higher intake category for energy and 18 out of 22 nutrients in FR-crude and FFQ-crude as compared to FR-crude and FFQ-adjusted, or FR-crude and FFQ-regressive (e.g., for fiber: 49% *vs.* 12% *vs.* 0%, respectively, Appendix B).

**Table 2:** Mean daily energy and nutrient intake by women (*n* 84) in the three-day unweighted food record (FR) and the food frequency questionnaire (FFQ).

Nutrient (unit)	FR-crude	FFQ-crude	*P* value	FFQ-adjusted	*P* value	FFQ-regressive	*P* value	FR-standardized	FFQ-standardized	*P* value
Energy (kcal)	1621.0	2740.5	<0.001	1621.0	0.792	1621.0 (1546.6, 1695.3)	0.181	2000.0	2000.0	-
	(1488.9, 1753.0)	(2501.9, 2979.1)		(1479.8, 1762.1)						
Total protein (g)	59.3	94.0	<0.001	55.6	0.030	59.3	0.242	76.0	69.7	<0.001
	(55.3, 63.4)	(86.1, 102.0)		(50.9, 60.3)		(57.5, 61.1)		(73.0, 79.0)	(67.2, 72.1)	
Animal protein (g)	38.5	60.1	<0.001	35.5	0.024	38.5	0.179	49.5	44.6	<0.001
								(46.7, 52.4)	(42.1, 47.1)	
	(35.6, 41.5)	(54.3, 65.8)		(32.1, 38.9)		(37.2, 39.8)				
Vegetable protein (g)	20.7	34.0	<0.001	20.1	0.256	20.7	0.145	26.3	25.1	0.117
	(19.1, 22.3)	(31.0, 36.9)		(18.3, 21.8)		(20.1, 21.3)		(25.1, 27.6)	(24.2, 25.9)	
Fat (g)	61.5	110.2	<0.001	65.2	0.157	61.5	0.070	73.0	79.2	0.001
	(54.8, 68.3)	(98.6, 121.7)		(58.3, 72.0)		(57.6, 65.4)		(69.7, 76.3)	(76.4, 82.0)	
SFA (g)	23.0	41.0	<0.001	24.2	0.265	23.0	0.086	27.4	29.4	0.013
	(20.4, 25.5)	(36.5, 45.4)		(21.6, 26.9)		(21.4, 24.5)		(26.1, 28.7)	(28.0, 30.8)	
MUFA (g)	24.6	44.5	<0.001	26.3	0.086	24.6	0.042	29.0	32.0	0.001
	(21.7, 27.5)	(39.7, 49.4)		(23.5, 29.2)		(23.1, 26.2)		(27.3, 30.8)	(30.7, 33.4)	
PUFA (g)	8.9	17.3	<0.001	10.3	0.002	8.9	0.026	10.5	12.5	<0.001
	(7.7, 10.0)	(15.4, 19.2)		(9.1, 11.4)		(8.3, 9.4)		(9.7, 11.4)	(11.9, 13.1)	
Cholesterol (mg)	294	310	0.117	183	<0.001	294	0.018	354	227	<0.001
								(324, 384)	(214, 239)	
	(258, 330)	(278, 341)		(165, 202)		(284, 304)				
Carbohydrates (g)	220.0	364.0	<0.001	215.3	0.258	220.0	0.189	275.7	266.5	0.046
	(203.3, 236.7)	(332.0, 396.0)		(196.5, 234.2)		(211.4, 228.6)		(269.1, 282.3)	(259.5, 273.5)	
Fibre (g)	15.9	30.2	<0.001	17.9	0.045	15.9	0.106	20.0	22.1	<0.001
	(14.4, 17.4)	(27.2, 33.2)		(16.1, 19.6)		(15.2, 16.5)		(18.9, 21.1)	(21.0, 23.3)	
Water (g)	1237	2180	<0.001	1290	0.971	1237	0.237	1640	1654	0.754
	(1160, 1315)	(1997, 2364)		(1181, 1398)		(1208, 1267)		(1525, 1756)	(1537, 1772)	
Sodium (mg)	1973	2510	<0.001	1485	<0.001	1973	0.124	2589	1851	<0.001
	(1820, 2127)	(2281, 2739)		(1349, 1620)		(1953, 1993)		(2396, 2783)	(1782, 1921)	
Potassium (mg)	2534	4496	<0.001	2660	0.586	2534	0.175	3226	3318	0.181
	(2332, 2736)	(4094, 4899)		(2422, 2897)		(2433, 2635)		(3058, 3394)	(3168, 3468)	
Calcium (mg)	601	1065	<0.001	630	0.786	601	0.128	769	800	0.524
	(537, 664)	(945, 1185)		(559, 701)		(568, 633)		(708, 829)	(724, 875)	
Phosphorus (mg)	999	1663	<0.001	983	0.366	999	0.207	1275	1236	0.149
	(922, 1076)	(1519, 1806)		(899, 1068)		(962, 1036)		(1214, 1336)	(1180, 1292)	
Magnesium (mg)	230	401	<0.001	237	0.775	230	0.159	294	297	0.336
	(211, 248)	(366, 436)		(216, 258)		(222, 237)		(277, 311)	(284, 310)	
Iron (mg)	8.7	14.5	<0.001	8.6	0.514	8.7	0.134	11.0	10.7	0.449
	(8.0, 9.4)	(13.2, 15.7)		(7.8, 9.3)		(8.4, 8.9)		(10.4, 11.6)	(10.3, 11.1)	
Zinc (mg)	7.7	12.6	<0.001	7.5	0.240	7.7	0.176	9.8	9.3	0.051
	(7.1, 8.2)	(11.5, 13.7)		(6.8, 8.1)		(7.5, 7.9)		(9.4, 10.2)	(9.0, 9.7)	
Copper (mg)	0.96	1.58	<0.001	0.93	0.378	0.96	0.131	1.21	1.16	0.209
	(0.87, 1.04)	(1.43, 1.72)		(0.85, 1.02)		(0.92, 0.99)		(1.14, 1.28)	(1.11, 1.21)	
Vitamin A (μg)	1069	1968	<0.001	1164	0.019	1069	0.001	1314	1434	0.003
	(853, 1285)	(1729, 2206)		(1023, 1305)		(1023, 1115)		(1047, 1582)	(1304, 1564)	
Retinol (μg)	457	802	<0.001	474	0.241	457	<0.001	576	574	0.138
	(279, 636)	(652, 951)		(386, 563)		(457, 458)		(331, 820)	(486, 662)	
β-carotene (μg)	3214	6989	<0.001	4134	<0.001	3214	<0.001	3921	5154	<0.001
	(2544, 3883)	(5978, 7999)		(3536, 4731)		(2925, 3502)		(3197, 4645)	(4510, 5797)	
Vitamin D (μg)	0.96	3.07	<0.001	1.82	<0.001	0.96	<0.001	1.35	2.18	<0.001
	(0.64, 1.28)	(2.63, 3.51)		(1.56, 2.08)		(0.96, 0.96)		(0.89, 1.81)	(1.99, 2.38)	
Vitamin E (mg)	7.8	15.8	<0.001	9.3	<0.001	7.8	0.029	9.2	11.5	<0.001
	(6.8, 8.8)	(14.3, 17.3)		(8.4, 10.3)		(7.2, 8.3)		(8.5, 10.0)	(11.1, 11.9)	
Vitamin B_1_ (mg)	1.02	1.61	<0.001	0.95	0.182	1.02	0.103	1.28	1.18	0.036
	(0.93, 1.11)	(1.47, 1.75)		(0.87, 1.04)		(0.99, 1.05)		(1.21, 1.35)	(1.15, 1.22)	
Vitamin B_2_ (mg)	1.30	2.17	<0.001	1.28	0.218	1.30	0.226	1.69	1.62	0.092
	(1.20, 1.40)	(1.97, 2.37)		(1.17, 1.40)		(1.26, 1.35)		(1.59, 1.79)	(1.52, 1.72)	
Niacin (mg)	12.1	19.5	<0.001	11.5	0.424	12.1	0.093	15.6	14.4	0.428
	(11.1, 13.2)	(17.8, 21.2)		(10.5, 12.5)		(11.8, 12.5)		(14.4, 16.8)	(13.9, 15.0)	
Vitamin B_6_ (mg)	1.44	2.55	<0.001	1.51	0.570	1.44	0·133	1.82	1.86	0.184
	(1.33, 1.56)	(2.32, 2.78)		(1.37, 1.64)		(1.39, 1.49)		(1.73, 1.92)	(1.8, 1.93)	
Folic acid (μg)	77	378	<0.001	224	<0.001	77	0.001	116	281	<0.001
	(64, 90)	(346, 411)		(205, 243)		(74, 80)		(92, 140)	(268, 294)	
Vitamin B_12_ (μg)	1.03	5.72	<0.001	3.38	<0.001	1.03	<0.001	1.67	4.18	<0.001
	(0.78, 1.28)	(5.01, 6.42)		(2.97, 3.79)		(1.00, 1.06)		(1.20, 2.14)	(3.83, 4.53)	
Vitamin C (mg)	97.8	207.0	<0.001	122.4	<0.001	97.8	0.020	122.5	151.0	<0.001
	(84.4, 111.3)	(182.8, 231.2)		(108.1, 136.8)		(91.2, 104.4)		(109.1, 136.0)	(138.2, 163.7)	

FR-crude—crude data from food records, FFQ-crude—crude data from FFQ, FFQ-adjusted—FFQ adjusted with β-coefficient, FFQ-regressive—FFQ adjusted by regression, FR-standardized—food records after energy standardization, FFQ-standardized—FFQ after energy standardization, *P* value—*t*-student test significance level after logarithmic transformation of data, NS—insignificant differences, ()—confidence interval for the mean value provided in brackets (95% CI), SFA—saturated fatty acids, MUFA—monounsaturated fatty acids, PUFA—polyunsaturated fatty acids.

**Table 3:** Comparison of energy and nutrient intake in women (*n* = 84) in the three-day unweighted food record (FR) and the food frequency questionnaire (FFQ).

Nutrient (unit)	FR-crude *vs.* FFQ-crude				FR-crude *vs.* FFQ-adjusted				FR-crude *vs.* FFQ-regressive				FR-standardized *vs.* FFQ-standardized			
	FFQ/FR (%)	*x* ± SD_difference _(LOA)	VC (%)	Bland-Altman Index (%)	FFQ/FR (%)	*x* ± SD_difference _(LOA)	VC (%)	Bland-Altman Index (%)	FFQ/FR (%)	*x* ± SD_difference _(LOA)	VC (%)	Bland-Altman Index (%)	FFQ/FR (%)	*x* ± SD_difference _(LOA)	VC (%)	Bland-Altman Index (%)
Energy (kcal)	179	1119.6 ± 908.6	42	6	106	0.0 ± 589.4	36	5	110	0.0 ± 502.8	31	5	100	0.0 ± 0.0	0	–
		(−661.3, 2900.5)				(−1155.3, 1155.3)				(−985.5, 985.5)				(0.0, 0.0)		
Total protein (g)	166	34.7 ± 32.8	43	4	98	−3.7 ± 21.3	37	4	107	0.0 ± 16.7	28	4	93	−6.3 ± 12.2	17	6
		(−29.6, 99.0)				(−45.5, 38.1)				(−32.7, 32.7)				(−30.2, 17.5)		
Animal protein (g)	167	21.5 ± 23.6	48	6	99	−3.0 ± 15.4	42	5	111	0.0 ± 12.1	31	5	94	−4.9 ± 12.3	26	5
		(−24.8, 67.8)				(−33.2, 27.2)				(−23.7, 23.7)				(−29.0, 19.2)		
Vegetable protein (g)	174	13.2 ± 12.8	47	6	103	−0.6 ± 8.8	43	4	110	0.0 ± 7.0	34	5	99	−1.3 ± 6.2	24	5
		(−11.9, 38.4)				(−17.8, 16.5)				(−13.8, 13.8)				(−13.5, 11.0)		
Fat (g)	209	48.6 ± 43.4	51	6	123	3.6 ± 28.7	45	6	122	0.0 ± 25.3	41	5	113	6.2 ± 17.8	23	6
		(−36.4, 133.6)														
						(−52.6, 59.8)				(−49.6, 49.6)				(−28.7, 41.0)		
SFA (g)	205	18.0 ± 16.4	51	4	121	1.3 ± 10.6	45	4	121	0.0 ± 9.2	40	4	112	2.0 ± 7.1	25	4
		(−14.2, 50.2)				(−19.4, 22.0)				(−18.1, 18.1)				(−12.0, 16.0)		
MUFA (g)	223	19.9 ± 18.9	55	6	132	1.7 ± 12.8	50	5	130	0.0 ± 11.3	46	4	119	3.0 ± 9.3	30	5
		(−17.2, 57.0)				(−23.5, 26.9)				(−22.2, 22.2)				(−15.2, 21.2)		
PUFA (g)	243	8.5 ± 7.7	58	6	144	1.4 ± 5.2	55	6	131	0.0 ± 4.6	51	2	132	1.9 ± 4.2	36	5
		(−6.6, 23.5)				(−8.9, 11.7)				(−8.9, 8.9)				(−6.2, 10.1)		
Cholesterol (mg)	143	16 ± 187	62	7	85	−111 ± 165	69	6	141	0 ± 160	54	6	77	−127 ± 158	54	8
		(−351, 383)				(−433, 212)				(−314, 314)				(−437, 183)		
Carbohydrates (g)	173	144.0 ± 126.1	43	7	102	−4.7 ± 81.3	37	7	109	0.0 ± 66.0	30	7	98	−9.3 ± 42.2	16	5
		(−103.0, 391.1)				(−164.0, 154.6)				(−129.4, 129.4)				(−92.1, 73.5)		
Fibre (g)	202	14.3 ± 12.3	53	5	119	2.0 ± 7.9	47	5	112	0.0 ± 6.1	38	6	114	2.1 ± 5.4	26	4
		(−9.7, 38.4)				(−13.5, 17.5)				(−12.0, 12.0)				(−8.4, 12.7)		
Water (g)	184	943 ± 782	46	7	109	52 ± 489	39	7	108	0 ± 328	27	7	108	14 ± 665	40	5
		(−589, 2475)				(−907, 1012)				(−643, 643)				(−1289, 1317)		
Sodium (mg)	140	537 ± 1192	53	6	83	−489 ± 880	51	5	112	0 ± 701	36	4	79	−738 ± 933	42	6
		(−1799, 2873)				(−2214, 1236)				(−1374, 1374)				(−2567, 1091)		
Potassium (mg)	188	1962 ± 1606	46	5	111	125 ± 1024	39	2	110	0 ± 806	32	5	106	92 ± 750	23	5
		(−1186, 5110)				(−1882, 2133)				(−1580, 1580)				(−1378, 1562)		
Calcium (mg)	196	464 ± 475	57	5	116	29 ± 307	50	5	115	0 ± 252	42	8	110	31 ± 323	41	5
		(−466, 1394)				(−574, 632)				(−493, 493)				(−603, 664)		
Phosphorus (mg)	176	664 ± 582	44	5	104	−16 ± 384	39	5	109	0 ± 314	31	4	99	−39 ± 229	18	6
		(−478, 1805)				(−768, 737)				(−615, 615)				(−487, 409)		
Magnesium (mg)	187	171 ± 148	47	6	110	8 ± 99	42	6	110	0 ± 79	34	5	105	3 ± 69	23	5
		(−118, 461)												(−131, 138)		
						(−186, 201)				(−155, 155)						
Iron (mg)	179	5.8 ± 5.5	48	6	106	−0.1 ± 3.8	44	6	111	0.0 ± 3.0	34	5	101	−0.3 ± 2.8	26	5
		(−5.0, 16.6)				(−7.5, 7.3)				(−5.9, 5.9)				(−5.9, 5.2)		
Zinc (mg)	174	4.9 ± 4.6	45	5	103	−0.2 ± 3.1	40	5	109	0 ± 2.4	31	4	98	−0.4 ± 1.9	20	5
		(−4.0, 13.9)				(−6.2, 5.8)				(−4.7, 4.7)				(−4.1, 3.2)		
Copper (mg)	177	0.62 ± 0.60	47	5	105	−0.02 ± 0.41	44	4	112	0.00 ± 0.35	37	5	99	−0.05 ± 0.27	23	4
		(−0.55, 1.79)				(−0.83, 0.78)				(−0.69, 0.69)				(−0.59, 0.48)		
Vitamin A (μg)	273	899 ± 1317	87	4	161	95 ± 1068	96	2	163	0 ± 974	91	2	148	120 ± 1318	96	1
		(−1683, 3481)				(−1998, 2188)				(−1908, 1908)				(−2464, 2703)		
Retinol (μg)	269	344 ± 1069	170	4	159	17 ± 915	196	2	173	0 ± 821	179	1	145	−2 ± 1199	209	1
		(−1751, 2440)				(−1776, 1810)				(−1609, 1609)				(−2353, 2349)		
β-carotene (μg)	447	3775 ± 4339	85	7	264	920 ± 3128	85	6	229	0 ± 2785	87	5	266	1233 ± 3416	75	6
		(−4729, 12,279)				(−5211, 7051)				(−5458, 5458)				(−5462, 7928)		
Vitamin D (μg)	1134	2.11 ± 2.49	124	4	670	0.86 ± 1.89	136	2	421	0.00 ± 1.48	154	2	668	0.83 ± 2.34	132	2
		(−2.77, 7.00)				(−2.85, 4.56)				(−2.90, 2.90)				(−3.76, 5.42)		
Vitamin E (mg)	263	8.0 ± 6.1	52	6	155	1.6 ± 4.3	50	6	134	0.0 ± 3.9	51	5	143	2.2 ± 3.5	34	5
		(−3.9, 20.0)				(−6.8, 10.0)				(−7.7, 7.7)				(−4.7, 9.2)		
Vitamin B_1_ (mg)	176	0.59 ± 0.62	47	5	104	−0.07 ± 0.44	45	6	116	0.00 ± 0.37	36	5	97	−0.09 ± 0.33	27	6
		(−0.63, 1.81)				(−0.93, 0.80)				(−0.73, 0.73)				(−0.74, 0.55)		
Vitamin B_2_ (mg)	174	0.87 ± 0.83	48	4	103	−0.02 ± 0.53	41	4	107	0.00 ± 0.40	31	4	98	−0.07 ± 0.45	27	4
		(−0.75, 2.49)				(−1.06, 1.02)				(−0.79, 0.79)				(−0.95, 0.80)		
Niacin (mg)	183	7.4 ± 7.7	48	2	108	−0.6 ± 5.5	46	5	117	0.0 ± 4.6	38	5	103	−1.2 ± 5.5	37	6
		(−7.6, 22.4)				(−11.3, 10.1)				(−9.0, 9.0)				(−11.9, 9.6)		
Vitamin B_6_ (mg)	192	1.11 ± 0.95	47	4	114	0.07 ± 0.61	42	2	114	0.00 ± 0.47	33	5	108	0.04 ± 0.47	26	6
		(−0.75, 2.96)				(−1.13, 1.27)				(−0.92, 0.92)				(−0.88, 0.97)		
Folic acid (μg)	1367	302 ± 174	77	2	808	147 ± 118	79	5	293	0 ± 56	73	5	828	165 ± 133	67	6
		(−41, 644)				(−85, 379)				(−110, 110)				(−95, 426)		
Vitamin B_12_ (μg)	4449	4.69 ± 3.55	105	7	2631	2.35 ± 2.34	106	7	743	0.00 ± 1.14	111	4	2581	2.51 ± 2.52	86	5
		(−2.27, 11.65)				(−2.24, 6.94)				(−2.24, 2.24)				(−2.43, 7.46)		
Vitamin C (mg)	260	109.2 ± 97.5	64	6	154	24.6 ± 64.6	59	7	133	0.0 ± 53.8	55	4	150	28.4 ± 64.6	47	5
		(−82.0, 300.3)				(−102.0, 151.2)				(−105.5, 105.5)				(−98.2, 155.1)		

FR-crude—crude data from food records, FFQ-crude—crude data from FFQ, FFQ-adjusted—FFQ adjusted with β-coefficient, FFQ-regressive—FFQ adjusted by regression, FR-standardized—food records after energy standardization, FFQ-standardized—FFQ after energy standardization; FFQ/FR (%)—mean nutrient intake quotient in the food frequency questionnaire (FFQ) and the three-day unweighted food record (FR), *x* ± SD_difference_—mean and standard deviation of the absolute difference (FFQ–FR), LOA—limits of agreement, *i.e.*, confidence interval for the difference between both methods (mean difference ± 1.96 SD_difference_), VC (%)—variation coefficient [(SD_difference_/*x*_both methods_) × 100%]; percentage of persons out of the limits of agreement LOA, SFA—saturated fatty acids, MUFA- monounsaturated fatty acids, PUFA—polyunsaturated fatty acids.

FR-crude—protein intake in food records (crude data), FFQ-crude—protein intake in FFQ (crude data), FFQ-adjusted—FFQ adjusted with β-coefficient, FFQ-regressive—FFQ adjusted by regression, FR-standardized—food records after energy standardization, FFQ-standardized—FFQ after energy standardization; Mean–mean protein intake for both methods (FFQ and FR).

**Table 4:** Comparison of the distribution of the percentage of women who failed to meet or met Polish dietary recommendations for energy and nutrients (*n* 84, % of the sample).

Nutrient	Women who failed to meet dietary recommendations				Women who met dietary recommendations			
	FR-crude	FFQ-crude	FFQ-adjusted	FFQ-regressive	FR-crude	FFQ-crude	FFQ-adjusted	FFQ-regressive
Energy ^A,B,C^	14 ^a,b^	26	77 ^a^	87 ^b^	2 ^a,b^	55 ^a^	10 ^b^	4
Total protein ^B^	0 ^a^	2	19 ^a^	0	13 ^a,b,c^	94 ^a^	57 ^b^	93 ^c^
Fat ^B^	14 ^a,b^	14	57 ^a^	65 ^b^	7 ^a,b^	70 ^a^	23 ^b^	10
SFA ^B,C^	10 ^a,b^	12	48 ^a^	49 ^b^	10 ^a,b,c^	75 ^a^	31 ^b^	21 ^c^
PUFA ^A,B,C^	52 ^a,b,c^	36 ^a^	71 ^b^	94 ^c^	4 ^a^	43 ^a^	10	1
Cholesterol	13 ^a,b,c^	42 ^a^	87 ^b^	26 ^c^	17 ^a^	29	6 ^a^	13
Carbohydrates	0 ^a^	0	5 ^a^	0	24 ^a,b,c^	99 ^a^	92 ^b^	100 ^c^
Fibre ^A,B^	37 ^a,b^	36	74 ^a^	96 ^b^	2 ^a,b^	50 ^a^	12 ^b^	0
Sodium	0 ^a,b^	8 ^a^	42 ^b^	0	5 ^a,b,c^	82 ^a^	29 ^b^	100 ^c^
Potassium ^A,B^	0 ^a,b,c^	48 ^a^	92 ^b^	100 ^c^	29 ^a,b^	32	4 ^a^	0 ^b^
Calcium ^A,B,C^	73 ^a,b^	40 ^a^	83	98 ^b^	4 ^a^	38 ^a^	8	1
Phosphorus ^B^	0 ^a^	1	5 ^a^	0	23 ^a,b,c^	98 ^a^	79 ^b^	100 ^c^
Magnesium ^B^	0 ^a,b,c^	11 ^a^	51 ^b^	52 ^c^	4 ^a,b^	75 ^a^	26 ^b^	8
Iron	0 ^a,b^	6 ^a^	42 ^b^	2	7 ^a,b,c^	88 ^a^	40 ^b^	37 ^c^
Zinc ^B,C^	0 ^a^	4	36 ^a^	2	10 ^a,b,c^	90 ^a^	39 ^b^	44 ^c^
Copper	0 ^a^	2	21 ^a^	0	10 ^a,b,c^	93 ^a^	56 ^b^	83 ^c^
Vitamin A	4	1	10	0	54 ^a,b,c^	98 ^a^	86 ^b^	100 ^c^
Vitamin E ^B,C^	0 ^a,b,c^	6 ^a^	35 ^b^	45 ^c^	10 ^a,b,c^	89 ^a^	46 ^b^	26 ^c^
Vitamin B_1_	0 ^a^	4	38 ^a^	2	13 ^a,b,c^	82 ^a^	40 ^b^	51 ^c^
Vitamin B_2_	0 ^a^	1	17 ^a^	0	13 ^a,b,c^	94 ^a^	65 ^b^	99 ^c^
Niacin	0 ^a,b^	5 ^a^	37 ^b^	2	6 ^a,b,c^	83 ^a^	38 ^b^	40 ^c^
Vitamin B_6_^B^	0 ^a^	2	19 ^a^	0	18 ^a,b,c^	94 ^a^	61 ^b^	83 ^c^
Vitamin C ^B^	0 ^a^	1	15 ^a^	1	36 ^a,b,c^	94 ^a^	76 ^b^	83 ^c^

FR-crude—crude data from food records, FFQ-crude—crude data from FFQ, FFQ-adjusted—FFQ adjusted with β-coefficient, FFQ-regressive—FFQ adjusted by regression; A, B, C—significance of differences at *p* < 0.05 between the distribution of the percentage of women in three intake category as follows: A: FR-crude and FFQ-crude, B: FR-crude and FFQ-adjusted, C: FR-crude and FFQ-regressive; ^a−a, b−b, c−c^—significance of differences at *p* < 0.05 between FR-crude and FFQ-crude or FFQ-adjusted or FFQ-regressive in pairs; SFA—saturated fatty acids, PUFA—polyunsaturated fatty acids.

### 3.2. Food Records (FR-Crude) *vs.* Food Frequency Questionnaire after Adjustment with Beta-Coefficient (FFQ-Adjusted)

The mean energy value of the diet after adjustment with beta-coefficient (FFQ-adjusted) was 1621.0 kcal (95% CI 1479.8, 1762.1 kcal), and it was equal to the mean energy value in FR-crude (Table 2). Significant differences in the mean content of thirteen nutrients were found between FR-crude and FFQ-adjusted.

The mean values of the FFQ-adjusted/FR-crude ratio for energy and eleven nutrients ranged from 90% to 110% (Table 3). The lowest values of the FFQ-adjusted/FR-crude ratio were obtained for sodium (83%) and cholesterol (85%), and the highest were for Vitamin B_12_ (2631%), folic acid (808%), and Vitamin D (670%).

The Bland-Altman index, not exceeding 5%, was obtained for energy and nineteen nutrients, in which Bland-Altman index equal to 2% was obtained for five nutrients: potassium, Vitamin A, retinol, Vitamin D, and Vitamin B_6_ (Table 3). A variation coefficient (VC) below 50% was obtained for energy and eighteen nutrients. The lowest values of VC were obtained for energy (36%), total protein and carbohydrates (37% each), and the highest was for retinol (196%), Vitamin D (136%), and Vitamin B_12_ (106%).

Distributions of the percentage of women in intake categories in FR-crude and FFQ-adjusted were significantly different for energy and thirteen nutrients (Table 4).

Adjustment with beta-coefficient of the mean intake of energy and nutrients significantly improved the compatibility of classification to the same intake category for fourteen nutrients in FR-crude and FFQ-adjusted as compared to FR-crude and FFQ-crude (e.g., for iron: 27% *vs.* 6%, respectively, Appendix B).

### 3.3. Food Records (FR-Crude) *vs.* Food Frequency Questionnaire after Adjustment with Regression Equations (FFQ-Regressive)

The mean energy value of the diet after regression adjustment (FFQ-regressive) was 1621.0 kcal (95% CI 1546.6, 1695.3 kcal), and it was equal to the mean energy value in FR-crude (Table 2). For FFQ adjusted by regression in comparison to FR-crude, significant differences were obtained for the mean intake of eleven nutrients: MUFA, PUFA, cholesterol, Vitamin A, retinol, β-carotene, Vitamin D, Vitamin E, folic acid, Vitamin B_12_, and Vitamin C (Table 2).

The mean values of FFQ-regressive/FR-crude ratio for all nutrients exceeded 100% (Table 3). The mean values of FFQ-regressive/FR-crude ratio for energy and ten nutrients ranged from 107% to 110%. The lowest values of FFQ-regressive/FR-crude ratio were obtained for total protein (107%) and Vitamin B_2_ (107%), and the highest for Vitamin B_12_ (743%), and Vitamin D (421%).

The Bland-Altman index not exceeding 5% was obtained for energy and 26 nutrients (Table 3). The lowest values of Bland-Altman index were obtained for retinol (1%) and PUFA, and Vitamin A and Vitamin D (2% each). A variation coefficient (VC) below 50% was obtained for energy (31%) and 21 nutrients. The lowest values of VC were obtained for water (27%), total protein (28%) and carbohydrates (30%), and the highest for retinol (179%), Vitamin D (154%), and Vitamin B_12_ (111%).

Distributions of the percentage of women in intake categories in FR-crude and FFQ-regressive were significantly different for energy and five nutrients (SFA, PUFA, calcium, zinc, and Vitamin E) (Table 4).

The application of regression analysis (FFQ-regressive) significantly improved compatibility classifying respondents to the same intake category for eighteen nutrients as compared to FFQ-crude and for eleven nutrients as compared to FFQ-adjusted (Appendix B). For example, more respondents were classified to the same carbohydrate intake category by FR-crude and FFQ-regressive than by FR-crude and FFQ-adjusted, or by FR-crude and FFQ-crude (24% *vs.* 6% *vs.* 4%, respectively).

### 3.4. Food Records after Standardization (FR-Standardized) *vs.* Food Frequency Questionnaire after Standardization (FFQ-Standardized)

After standardization of the energy value of diets to 2000 kcal, significant differences between FR-standardized and FFQ-standardized were obtained for eighteen nutrients (Table 2).

The mean values of FFQ-standardized/FR-standardized ratio for fifteen nutrients ranged from 90% to 110% (Table 3). The lowest values of FFQ-standardized/FR-standardized ratio were obtained for cholesterol (77%) and sodium (79%), and the highest for Vitamin B_12_ (2581%), folic acid (828%), and Vitamin D (668%).

The Bland-Altman index not exceeding 5% was obtained for 21 nutrients (Table 3). The lowest values of Bland-Altman index were obtained for Vitamin A and retinol (1% each) and for Vitamin D (2%). A variation coefficient (VC) below 50% was obtained for 24 nutrients. The lowest values of VC were obtained for carbohydrates (16%), total protein (17%), and phosphorus (18%), and the highest was for retinol (209%) and Vitamin D (132%).

**Figure 2:**
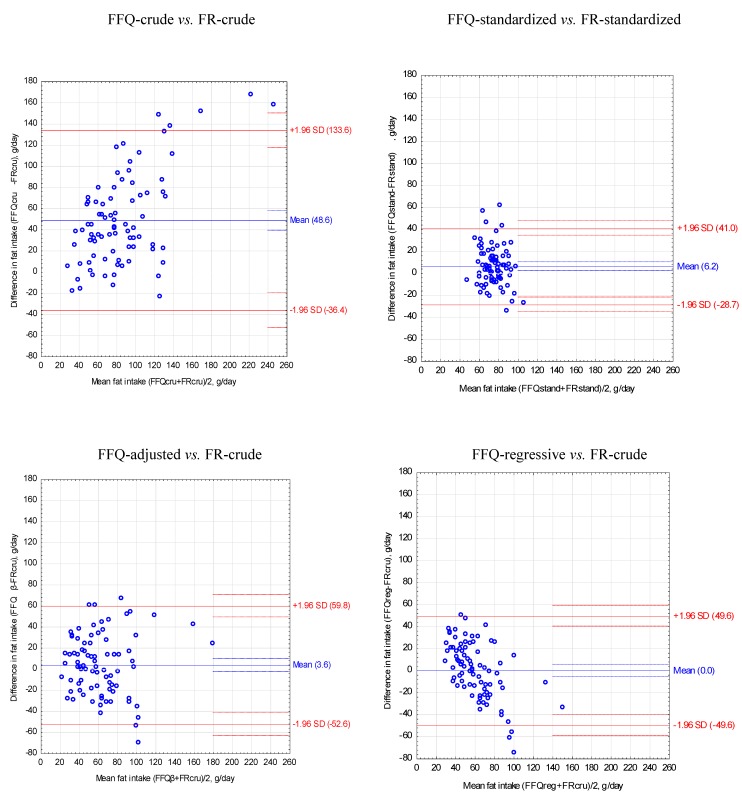
Bland-Altman plots to assess agreement between the food frequency questionnaire (FFQ) and three-day unweighted food record (FR) for fat intake in 84 women.

**Figure 3:**
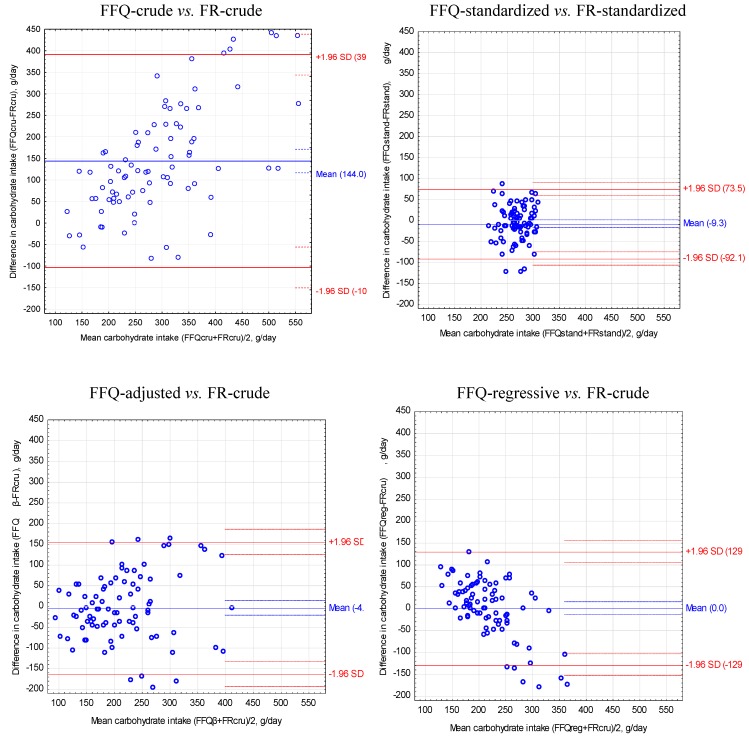
Bland-Altman plots to assess agreement between the food frequency questionnaire (FFQ) and three-day unweighted food record (FR) for carbohydrates intake in 84 women.

For FFQ and food record after standardization of the energy value of diets to 2000 kcal obtained the narrowest limits of agreement (LOA) and the lowest values of VC compared to other methods of adjustment of energy and nutrient intake (Table 3, Figure 1, Figure 2, Figure 3).

FR-crude—fat intake in food records (crude data), FFQ-crude—fat intake in FFQ (crude data), FFQ-adjusted—FFQ adjusted with β-coefficient, FFQ-regressive—FFQ adjusted by regression, FR-standardized—food records after energy standardization, FFQ-standardized—FFQ after energy standardization; Mean—mean fat intake for both methods (FFQ and FR).

FR-crude—carbohydrates intake in food records (crude data), FFQ-crude—carbohydrates intake in FFQ (crude data), FFQ-adjusted—FFQ adjusted with β-coefficient, FFQ-regressive—FFQ adjusted by regression, FR-standardized—food records after energy standardization, FFQ-standardized—FFQ after energy standardization; Mean—mean carbohydrates intake for both methods (FFQ and FR).

## 4. Discussion

### 4.1. Differences in Energy and Nutritional Value of Diet Obtained by FFQ and Food Record

The research proved overestimation of intake by the FFQ as compared to the three-day unweighted food record (crude data) for energy by 69% and many nutrients as follows: total protein, animal protein, vegetable protein, fat, saturated fatty acids (SFA), monounsaturated fatty acids (MUFA), polyunsaturated fatty acids (PUFA), carbohydrates, fiber, water, sodium, potassium, calcium, phosphorus, magnesium, iron, zinc, copper, Vitamin A, retinol, β-carotene, Vitamin D, Vitamin E, Vitamin B_1_, Vitamin B_2_, niacin, Vitamin B_6_, folic acid, Vitamin B_12_, and Vitamin C. Numerous studies reported higher energy and nutritional value of diets assessed by FFQ in comparison to other nutritional assessment methods [6,11,12,13,14,15,16]. Brunner *et al.* [6] demonstrated in women that the mean energy value of diets was about 10% higher if the FFQ was used as compared to seven-day unweighted food records. Those authors did not found any differences in the energy value of men’s diets. For women, Goulet *et al.* [7] found an overestimation of intake of total fat and monounsaturated fatty acids, and underestimation of fiber intake with the use of the FFQ in comparison to three-day unweighted food records.

In our study, the overestimation of energy and nutrient intake by the FFQ may be related to: (i) the respondent, e.g., due to inadequate estimate the food portion size, social desirability bias or social approval bias, (ii) the interviewer, e.g., because of bias related to recipe of dishes or the use of the “Food Composition Table”, (iii) the questionnaire structure, e.g., because of a long food list, many separate questions concerning the intake of vegetables and fruit were listed separately and in dishes [1,2,4,5,10]. For example, we stated the higher intake of fiber, β-carotene, folic acid, and Vitamin C obtained from the FFQ. This may result from overestimation of fruit and vegetable intake caused by the questionnaire structure and/or overestimation of the intake of foods considered as “healthy” food [1,4,5,10]. Similarly, the FFQ used by Brunner *et al.* [6] tended to overestimate the intake of plant origin nutrients (e.g., carotene) and the authors suspect it could be connected with the seasonal intake of some foods (e.g., vegetables, fruit) and many items to assess vegetable intake on the questionnaire. This was confirmed by Ambrosini *et al.* [23]. In our FFQ, there were separate questions for fruit and vegetables eaten in different seasons and for low- and high-fat foods. However, we found a higher intake of total fat, MUFA, PUFA, and fat-soluble Vitamins (A, β-carotene, D, E) and lower intake of cholesterol, total protein, and animal protein when the intake was assessed by the FFQ, regardless of energy adjustment. This suggests that overestimation of intake was more probable for plant origin fats than animal origin fats. Overestimation of the plant origin fat intake (e.g., vegetable oils) and underestimation of the dietary sources of animal fats (e.g., meat with a high fat content) obtained by our FFQ may also be caused by a social approval bias (trend to respond to obtain approval) or a social desirability bias (trend to respond to avoid criticism) in the women [1,5].

Our results suggest possible difficulties in assessing the food portion size consumed by respondents. This is consistent with the observations made by other authors [1,32]. This can be proven by obtaining a similar nutritional value of diets by the FFQ and food records after adjusting for the energy and nutrient intake in the FFQ. An “album of photographs of food products and dishes” [24] was used in the FFQ and food records containing photographs of three different portion sizes of a given food or dish (small, medium and large). Nelson *et al.* [32] found that the portion sizes estimated on the basis of several photographs differed on average by −4% to +5% from the real portion size of the food. As results from numerous studies, the size of error depends on respondent characteristics (e.g., age, sex, BMI) and their ability to adequately assess the portion size [1,32]. Respondents usually have more difficulty in estimating the portion sizes of food with irregular shapes and light products of considerable volume (e.g., lettuce), but they are better at estimating the portion sizes of liquid products (e.g., milk) [1,8]. For example, respondents using photos of different serving sizes considerably overestimated the amount of consumed butter and margarine [32]. This can partly explain the differences in the intake of fat and fat-soluble Vitamins recorded in our study, observed regardless of the energy adjustment applied for FFQ.

### 4.2. Differences in Nutritional Value of Diet after Energy Adjustment

Adjustment of energy and nutrient intake performed for our FFQ by various methods improved the agreement for energy and nutritional value of diet obtained by FFQ and three-day unweighted food records. The most compatible results were obtained after adjusting energy and nutrient intake from the FFQ by regression equations, both at the group and individual level of dietary assessment.

On the group level of dietary assessment, after adjusting energy and nutrient intake by various methods, the following nutrient intakes were still overestimated: fat, SFA, MUFA, PUFA, fiber, Vitamin A, β-carotene, Vitamin D, Vitamin E, folic acid, Vitamin B_12_, and Vitamin C, but intake of total protein, animal protein, cholesterol, carbohydrates, sodium, zinc, and Vitamin B_1_ were underestimated by the FFQ compared to the food record. The best results were obtained for the FFQ adjusted by regression, for which differences in the mean intake were observed only for following nutrients: MUFA, PUFA, cholesterol, Vitamin A, retinol, β-carotene, Vitamin D, Vitamin E, folic acid, Vitamin B_12_, and Vitamin C, but any nutrient intake was underestimated. Fialkowski *et al.* [11] for the FFQ after energy adjustment compared to the food record, for women stated overestimation of the mean intake of: total protein, total fat, MUFA, PUFA, Vitamin C, and underestimation of intake of cholesterol, folic acid, and iron.

On the individual level of dietary assessment, the adjustment of energy and nutrient intake applied to the FFQ improved the ability of the questionnaire to classify respondents to the same intake category. The highest compatibility of respondent classification to the same intake category was obtained by the regression-adjusted FFQ and food record. For example, over 70% respondents were compatibly classified for potassium and calcium and over 60% respondents for iron and zinc. Brunner *et al.* [6] also reported classifying a higher number of respondents to the same intake category by FFQ after energy adjustment and seven-day unweighted food record, and reducing the degree of misclassification into extreme quartiles of intake for most nutrients.

Differences in energy and nutritional value of diet between FFQ and food records, observed regardless of the energy adjustment applied for FFQ, could result from intra- and inter-subject variability [1]. Values of variation coefficient (VC) indicate low variability in the intake of total protein and carbohydrates and a high variability in the intake of Vitamin A, retinol, β-carotene, Vitamin D, Vitamin B_12_, and cholesterol, which is compatible with many papers [1,13,33]. A high variability in the β-carotene intake can be related to seasonal variability in the consumption of vegetables and fruits, while a high variability in the intake of cholesterol, Vitamin A and Vitamin B_12_ may result from the high intra- and inter-subject variability in the intake of offal (e.g., liver) which are a rich, although relatively rarely-consumed, source of those nutrients [1,13,33].

The wide limits of agreement observed in our study for crude data obtained by food record and the FFQ may indicate that the sample was small or/and the variability of results was large [30]. Generally, the energy adjustment performed for our FFQ resulted in decreasing the variability of the nutrient intake, which is shown by the narrower limits of agreement (LOA) and the lower values of variation coefficient (VC). This was particularly evident for the FFQ after standardization of the energy value of diets to 2000 kcal and, to a lesser extent, for the FFQ adjusted by regression. The reason may be that standardization for energy intake results in decreased variability in nutrient intake [1].

On the individual level of dietary assessment, for the FFQ adjusted by regression and food record in comparison to crude data from both methods, we did not reveal better compatibility of respondent classification to the same intake category for total fat, cholesterol, sodium, and Vitamin B_2_. Consequently, particular care should be taken while interpreting the results for nutrients characterized by considerable intake variability.

The regression analysis also seems to be the best method of adjustment of energy and nutrient intake in order to assess the respondent diet in relation to the dietary recommendations. After adjustment of energy and nutrients intake, we found more women who failed to meet dietary recommendations in comparison to the crude data from the FFQ. This was observed to a smaller extent for the FFQ adjusted by regression than the FFQ adjusted by a beta-coefficient. The results of the dietary assessment made by adjusted FFQ are compatible with many Polish papers assessing nutrition of Poles [34]. It was revealed that the diet of Polish women is too low in fiber, potassium, zinc, copper, magnesium, calcium, iron, folic acid, Vitamin B, Vitamin C, Vitamin A, Vitamin E, and PUFA [34]. Using regression-adjusted FFQ, we confirm the excessively low intake of fat, SFA, PUFA, cholesterol, fiber, potassium, calcium, magnesium, and Vitamin E.

### 4.3. Study Limitations

Eight percent of respondents were on a regular diet and 23% of them taking care of slim figure. Although being on a regular diet made it difficult to compare methods, this was declared by women in the FFQ as well as the three-day unweighted food record. Thus it was assumed that being on a regular diet in the same way resulted on a dietary assessment of both methods and did not affect a comparison of the results.

The next limitation of our study and cause of differences in the intake of energy and nutrients found between both methods could be the conversion algorithms used for the FFQ. The algorithm was designed to convert the recipes of dishes into components (single foods). Some foods were substituted by foods with similar composition due to the limited amount of food provided in the “Food composition tables” [25]. Although this procedure is often unavoidable in dietary assessment, it is a cause of bias [1,10].

Furthermore, the short-term unweighted food record is not an ideal “gold standard” when compared with a long-term FFQ, since the three-day food record does not cover food intake over the year like the FFQ. Obviously, it would be better to repeat the food record several times during the year (e.g., in each season) [10]. However, the aim of our study was to compare both methods and find the best way to adjust the results of both methods and prepare one well-done interpretation. Secondly, the food record provides more detailed information about food recipes and intake of specific foods (e.g., novel food), which were not included in the list of products in FFQ [1,35], and may expose the differences between the results obtained by both methods.

### 4.4. Study Strengths

The beneficial feature of our study is using many adjustment and statistical techniques to compare the FFQ and three-day unweighted food record. We compared mean values and distributions and used the Bland-Altman method, which is the most recommended validation procedure [10,23]. This allowed creation of strong conclusions regarding group and individual differences between the FFQ and three-day unweighted food record. We used several methods for adjusting the FFQ, from a simple beta-coefficient method to regression equations made for each nutrient separately, to select the best method of adjustment.

The regression equations in this study will allow other researchers to adapt the results obtained by the FFQ to results obtained by the food record. This will allow the use of both methods simultaneously in a dietary assessment and prepare a single, comprehensive interpretation.

## 5. Conclusions

It was proven that the application of various methods of adjusting energy and nutrient intake to different degrees reduced the differences in energy and nutritional value between the food frequency questionnaire and three-day unweighted food records. Adjustment of energy and nutritional value applied for the FFQ significantly improved the agreement between results obtained by the FFQ and the food record, both in the individual and group level dietary assessments. The application of the regression equations for energy and nutrients in the paper will allow other researchers to accurately approximate the results obtained from the FFQ to the results from the unweighted food record and the combined analysis of results of some of the most common methods of nutritional assessment.

Improving compatibility in the assessment of the nutritional value of the diet obtained by the food record and the FFQ after energy adjustment suggests that the FFQ is better at reproducing the composition and the quality of diet of women and worse in representing the amount of consumed food. To conclude, differences in the nutritional values of diets assessed by both methods result largely from an overestimation of the energy intake by the FFQ and probable difficulties in assessing the portion size of consumed food by women. The use of a food frequency questionnaire requires particular care from the researchers while collecting information on the amount of consumed food. The results obtained and the conclusions drawn are limited to women and may be inapplicable to men.

## Appendix

**Appendix A:** Regression equations for energy and nutrient intake obtained by the FFQ. Formula: *y* = *ax* + *b* ± estimation error; *x*—nutrient intake according to crude data from FFQ (FFQ-crude); *y*—nutrient intake in crude data from FFQ adjusted by regression to data from three-day unweighted food record (FFQ-regressive).

Nutrient	*a*	*b*	Estimation error	Regression equations	*R*^2^ corrected	*P*
Energy (kcal)	0.31	766.77	505.85	*y* = 0.31 × *x* + 766,77 ± 505.85	0.31	<0.001
Total protein (g)	0.23	37.72	16.77	*y* = 0.23 × *x* + 37.72 ± 16.77	0.19	<0.001
Animal protein (g)	0.23	24.72	12.14	*y* = 0.23 × *x* + 24.72 ± 12.14	0.19	<0.001
Vegetable protein (g)	0.21	13.75	7.07	*y* = 0.21 × *x* + 13.75 ± 7.07	0.12	<0.001
Fat (g)	0.34	24.29	25.45	*y* = 0.34 × *x* + 24.29 ± 25.45	0.33	<0.001
SFA (g)	0.34	8.97	9.27	*y* = 0.34 × *x* + 8.97 ± 9.27	0.36	<0.001
MUFA (g)	0.32	10.39	11.40	*y* = 0.32 × *x* + 10.39 ± 11.40	0.27	<0.001
PUFA (g)	0.30	3.74	4.59	*y* = 0.30 × *x* + 3.74 ± 4.59	0.23	<0.001
Cholesterol (mg)	0.32	193.63	160.92	*y* = 0.32 × *x* + 193.63 ± 160.92	0.07	<0.05
Carbohydrates (g)	0.27	122.05	66.43	*y* = 0.27 × *x* + 122.05 ± 66.43	0.25	<0.001
Fibre (g)	0.23	9.02	6.14	*y* = 0.23 × *x* + 9.02 ± 6.14	0.20	<0.001
Water (g)	0.16	882.25	330.08	*y* = 0.16 × *x* + 882.25 ± 330.08	0.14	<0.001
Sodium (mg)	0.09	1752.39	705.34	*y* = 0.09 × *x* + 1752.39 ± 705.34	0.01	NS
Potassium (mg)	0.25	1406.89	810.86	*y* = 0.25 × *x* + 1406.89 ± 810.86	0.24	<0.001
Calcium (mg)	0.27	311.23	253.14	*y* = 0.27 × *x* + 311.23 ± 253.14	0.25	<0.001
Phosphorus (mg)	0.26	572.36	315.71	*y* = 0.26 × *x* + 572.36 ± 315.71	0.22	<0.001
Magnesium (mg)	0.23	139.14	79.58	*y* = 0.23 × *x* + 139.14 ± 79.58	0.16	<0.001
Iron (mg)	0.20	5.82	3.01	*y* = 0.20 × *x* + 5.82 ± 3.01	0.11	<0.01
Zinc (mg)	0.20	5.11	2.42	*y* = 0.20 × *x* + 5.11 ± 2.42	0.13	<0.001
Copper (mg)	0.26	0.54	0.35	*y* = 0.26 × *x* + 0.54 ± 0.35	0.18	<0.001
Vitamin A (μg)	0.19	690.11	979.55	*y* = 0.19 × *x* + 690.11 ± 979.55	0.03	NS
Retinol (μg)	0.01	452.78	825.83	*y* = 0.01 × *x* + 452.78 ± 825.83	–0.01	NS
β-carotene (μg)	0.29	1217.87	2801.64	*y* = 0.29 × *x* + 1217.87 ± 2801.64	0.18	<0.001
Vitamin D (μg)	0.01	0.93	1.49	*y* = 0.01 × *x* + 0.93 ± 1.49	–0.01	NS
Vitamin E (mg)	0.34	2.39	3.94	*y* = 0.34 × *x* + 2.39 ± 3.94	0.26	<0.001
Vitamin B_1 _(mg)	0.23	0.65	0.37	*y* = 0.23 × *x* + 0.65 ± 0.37	0.13	<0.001
Vitamin B_2_ (mg)	0.22	0.83	0.41	*y* = 0.22 × *x* + 0.83 ± 0.41	0.19	<0.001
Niacin (mg)	0.21	8.07	4.60	*y* = 0.21 × *x* + 8.07 ± 4.60	0.10	<0.01
Vitamin B_6 _(mg)	0.22	0.88	0.47	*y* = 0.22 × *x* + 0.88 ± 0.47	0.19	<0.001
Folic acid (μg)	–0.10	115.60	56.45	*y* = –0.10 × *x* + 115.60 ± 56.45	0.06	<0.05
Vitamin B_12_(μg)	–0.04	1.27	1.15	*y*= –0.04 × *x* + 1.27 ± 1.15	0.00	NS
Vitamin C (mg)	0.27	41.46	54.17	*y* = 0.27 × *x* + 41.46 ± 54.17	0.23	<0.001

*R*^2^ corrected—determination coefficient, *p* value—*t*-student test significance level, NS—insignificant differences, SFA—saturated fatty acids, MUFA—monounsaturated fatty acids, PUFA—polyunsaturated fatty acids.

**Appendix B:** Agreement between classification into the energy and nutrient category in the three-day unweighted food record (FR) and the food frequency questionnaire (FFQ) in women (*n* 84, % of the sample).

Nutrient	Compatible classification of FR-crude and FFQ			FFQ classifies below FR-crude			FFQ classifies above FR-crude		
	FFQ-crude	FFQ-adjusted	FFQ-regressive	FFQ-crude	FFQ-adjusted	FFQ-regressive	FFQ-crude	FFQ-adjusted	FFQ-regressive
Energy	26	27	26	18 ^a,b^	64 ^a^	73 ^b^	56 ^a,b^	8 ^a,c^	1 ^b,c^
Total protein	6 ^a,b^	30 ^a^	20 ^b^	2 ^a^	13 ^a,b^	0 ^b^	92 ^a,b^	57 ^a,c^	80 ^b,c^
Fat	21 ^a^	35 ^a^	33	10 ^a,b^	45 ^a^	57 ^b^	69 ^a,b^	20 ^a,c^	10 ^b,c^
SFA	18 ^a,b^	33 ^a^	35 ^b^	7 ^a,b^	37 ^a^	46 ^b^	75 ^a,b^	30 ^a,c^	19 ^b,c^
PUFA	32 ^a^	33 ^b^	56 ^a,b^	26 ^a,b^	58 ^a,c^	43 ^b,c^	42 ^a,b^	8 ^a,c^	1 ^b,c^
Cholesterol	38 ^a^	21 ^a,b^	52 ^b^	33 ^a^	74 ^a,b^	31 ^b^	29 ^a,b^	5 ^a,c^	17 ^b,c^
Carbohydrates	4 ^a^	6 ^b^	24 ^a,b^	0	4	0	96 ^a^	90 ^b^	76 ^a,b^
Fibre	25 ^a^	27	40 ^a^	26 ^a,b^	61 ^a^	60 ^b^	49 ^a,b^	12 ^a,c^	0 ^b,c^
Sodium	11 ^a^	39 ^a,b^	5 ^b^	6 ^a,b^	32 ^a,c^	0 ^b,c^	83 ^a,b^	29 ^a,c^	95 ^b,c^
Potassium	31 ^a,b^	18 ^a,c^	71 ^b,c^	37 ^a,b^	79 ^a,c^	0 ^b,c^	32 ^a^	4 ^a,b^	29 ^b^
Calcium	30 ^a^	20 ^b^	73 ^a,b^	32 ^a^	73 ^a,b^	26 ^b^	38 ^a,b^	7 ^a,c^	1 ^b,c^
Phosphorus	4 ^a,b^	15 ^a^	23 ^b^	1	5 ^a^	0 ^a^	95 ^a,b^	80 ^a^	77 ^b^
Magnesium	13 ^a,b^	31 ^a^	38 ^b^	10 ^a,b^	42 ^a,c^	55 ^b,c^	77 ^a,b^	27 ^a,c^	7 ^b,c^
Iron	6 ^a,b^	27 ^a,c^	63 ^b,c^	6 ^a^	32 ^a,b^	5 ^b^	88 ^a,b^	40 ^a^	32 ^b^
Zinc	7 ^a,b^	33 ^a,c^	61 ^b,c^	4 ^a^	27 ^a,b^	4 ^b^	89 ^a,b^	39 ^a^	36 ^b^
Copper	6 ^a,b^	29 ^a^	26 ^b^	2 ^a^	15 ^a,b^	0 ^b^	92 ^a,b^	56 ^a,c^	74 ^b,c^
Vitamin A	4 ^a^	7 ^b^	54 ^a,b^	1 ^a^	8 ^a,b^	0 ^b^	95 ^a,b^	85 ^a,c^	46 ^b,c^
Vitamin E	8 ^a,b^	27 ^a^	31 ^b^	5 ^a,b^	26 ^a,c^	49 ^b,c^	87 ^a,b^	46 ^a,c^	20 ^b,c^
Vitamin B_1_	12 ^a,b^	31 ^a,c^	55 ^b,c^	4 ^a^	29 ^a,b^	5 ^b^	85 ^a,b^	40 ^a^	40 ^b^
Vitamin B_2_	6 ^a^	20 ^a^	14	1 ^a^	13 ^a,b^	0 ^b^	93 ^a^	67 ^a,b^	86 ^b^
Niacin	10 ^a,b^	33 ^a,c^	56 ^b,c^	5 ^a^	27 ^a,b^	6 ^b^	86 ^a,b^	39 ^a^	38 ^b^
Vitamin B_6_	6 ^a,b^	25 ^a^	35 ^b^	2 ^a^	13 ^a,b^	0 ^b^	92 ^a,b^	62 ^a^	65 ^b^
Vitamin C	7 ^a^	11 ^b^	46 ^a,b^	1 ^a^	13 ^a,b^	4 ^b^	92 ^a,b^	76 ^a,c^	50 ^b,c^

FR-crude—crude data from food records, FFQ-crude—crude data from FFQ, FFQ-adjusted—FFQ adjusted with β-coefficient, FFQ-regressive—FFQ adjusted by regression; ^a−a, b−b, c−c^–significance of differences at *p* < 0.05 between the FR-crude and the FFQ-crude or FFQ-adjusted or FFQ-regressive; SFA—saturated fatty acids, PUFA—polyunsaturated fatty acids.
